# Predictors of Return to Sports Following the Modified Broström Procedure for Chronic Ankle Instability

**DOI:** 10.3390/jcm14176046

**Published:** 2025-08-26

**Authors:** Sung-Hoo Kim, Seung-Myung Choi, Byung-Ki Cho

**Affiliations:** 1 Department of Orthopaedic Surgery, Chungbuk National University Hospital, Cheongju 28644, Republic of Korea; hoo414414@hanmail.net; 2 Department of Orthopaedic Surgery, Daejeon Bon Hospital, Daejeon 34188, Republic of Korea; davidchoi1530@gmail.com; 3 Department of Orthopaedic Surgery, College of Medicine, Chungbuk National University, Cheongju 28644, Republic of Korea

**Keywords:** ankle, chronic instability, modified Broström procedure, return to sports

## Abstract

**Background/Objectives**: Despite a substantial duration of recovery following the modified Broström procedure (MBP), many individuals do not entirely recover their preinjury sports performance. Return to sports (RTS) can be affected by multiple elements apart from a patient’s motivation. This study aimed to investigate the factors influencing RTS after anatomical ligament repair for chronic ankle instability. **Methods**: Sixty-two patients aged under 35 who underwent the MBP were regularly monitored for up to 3 years. Of these, 51 patients (82.3%) returned to their preinjury level of sports activity (return group), while 11 patients (17.7%) complained of partial or significant limitations (non-return group). Clinical outcomes were measured by the Foot and Ankle Outcome Score (FAOS) and the Foot and Ankle Ability Measure (FAAM). Mechanical stability was examined through physical examination and stress radiography. Peroneal strength was evaluated with an isokinetic dynamometer. Static and dynamic postural control abilities were tested using Biodex posturography. **Results**: Significant group differences were found in FAOS pain (94.7 points in the return group vs. 85.1 points in the non-return group; *p* = 0.004) and sports (91.2 vs. 78.8 points; *p* < 0.001) subscales. In the FAAM, the sports activity subscale also showed significant disparities (90.5 vs. 77.4 points, *p* < 0.001). Mechanical instability recurred in 2 patients (3.9%) in the return group and 4 patients (36.4%) in the non-return group, indicating a significant difference (*p* < 0.001). No notable differences were identified in stress radiography values or peroneal strength measurements. Posturographic evaluation showed that static postural control ability (overall stability index) did not differ significantly between the groups (1.22 in the return group vs. 1.43 in the non-return group); however, dynamic postural control ability differed substantially (1.41 vs. 2.33, *p* = 0.002). **Conclusions**: Residual pain, recurrence of mechanical instability, and insufficient recovery of dynamic postural control ability were associated with the return to preinjury level of sports activity after the MBP. Comprehensive rehabilitation protocols should address these factors to facilitate optimal postoperative sports participation.

## 1. Introduction

Chronic lateral ankle instability (CLAI) is one of the most frequent complications following ankle sprain injuries [1,2,3,4]. With continuous modification using recent emerging techniques, the Broström repair procedure provides restoration of reliable stability and satisfactory clinical outcomes, as a gold-standard anatomical ligament repair surgery for CLAI [1,3,5,6]. However, even after an extended period following the modified Broström procedure (MBP), a substantial proportion of patients appear to reduce their sports participation and cease sports activities prematurely. Maffulli et al. reported that a large proportion (42%) of patients experienced reduced sports activity during long-term follow-up after the MBP [7]. There is currently no consensus regarding the factors contributing to failure to resume the previous level of sports activity after surgical treatment. Return to sports (RTS) may be affected by several variables apart from patient motivation (intention to RTS), such as persistent pain or swelling, insufficient restoration of mechanical stability, weakness of peroneal strength, deficiencies in postural or neuromuscular control, and impaired proprioception [8,9,10,11,12].

The RTS rate serves as a meaningful indicator of functional outcome following operative treatment and provides a useful reference during patient counseling for CLAI. Successful return to preinjury level of sports participation represents a key determinant of patient satisfaction after surgical intervention for CLAI. This consideration is pertinent both for professional athletes and for those engaged in recreational sports. We hypothesized that there would be specific factors that make the difference between patients with and without RTS after the MBP. This comparative study was designed to identify the factors affecting the return to sports activity after anatomical ligament repair for CLAI.

## 2. Materials and Methods

### 2.1. Study Design and Subjects

This research was conducted as a single-center longitudinal cohort study employing time series analysis. All the data were collected prospectively and analyzed retrospectively. The study protocol and related investigations received approval from the Institutional Review Board. Patients were recruited for eligibility after electing to undergo surgical treatment (MBP) with a diagnosis of CLAI. All eligible patients received informed consent regarding the postoperative visit schedule and the nature of examinations. The informed consent also stated that refusal or withdrawal from the study would not result in any disadvantages for participants.

**Figure 1:**
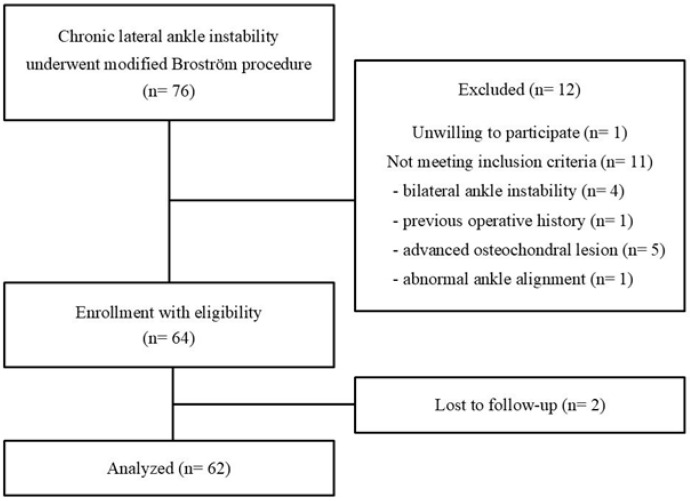
Consort flowchart diagram of this study.

Between January 2020 and December 2021, a total of 76 patients (80 ankles) consecutively underwent the MBP for CLAI after experiencing at least 3 months of unsuccessful rehabilitation. Of these patients, 64 patients (64 ankles) who met the inclusion criteria were enrolled in this study. Ultimately, 62 patients (62 ankles) with a minimum follow-up period of 3 years postoperatively were included in the analysis (Figure 1). The inclusion criteria for this study were as followings: (1) patients under 35 years of age with intention to return to sports, (2) unilateral ankle instability, (3) no previous ankle ligament or fracture surgery history, (4) absence of advanced osteochondral pathology requiring bone marrow stimulation or cartilage transplantation, (5) no hindfoot varus or equinus deformity requiring additional corrective procedures, and (6) no visual, vestibular, or neuromuscular impairments. The scheduled postoperative follow-up intervals were 1 month prior to surgery, and then 6 months, 1 year, 2 years, and 3 years postoperatively. Patients were considered compliant with the follow-up schedule if they presented within 1 month before or after the designated time point. Regardless of the timing of return to sports activity, all the participants were classified as either in the return group or non-return group based on whether they achieved preinjury sports performance. Because this study included patients with various previous levels of sports activity, we thought there was clear heterogeneity on capability levels needed for sports activity. A return to preinjury level of sports activity was determined dependent on patient self-report instead of standardized tools or objective performance metrics. Among the 62 patients analyzed, 51 patients (82.3%) successfully resumed their preinjury level of sports activity (return group), while 11 (17.7%) continued to experience partial or substantial limitations (non-return group).

The mean age of the study population was 27.1 years (range: 18 to 35 years), and the mean duration of symptoms was 33.8 months (range: 10 to 64 months). There were 38 male and 24 female patients in the cohort. Initial injuries were attributed to sports-related incidents in 36 cases, slip and fall during walking in 24 cases, and traffic accidents in 2 cases. Eleven of the 62 patients were professional or junior level athletes, and others were participants in recreational or amateur levels of sports activities.

### 2.2. Rehabilitation Protocol Following Surgery

Initially, all the patients underwent arthroscopic examination and simple procedures, including synovectomy, chondral debridement, and loose body removal. The MBP was performed in a standard fashion by a single senior surgeon. Both the anterior talofibular (ATF) and calcaneofibular (CF) ligaments were repaired using suture anchors, and the inferior extensor retinaculum was imbricated over the repaired lateral ligaments.

A short-leg cast was applied, and partial weight-bearing ambulation with crutches was maintained for 3 weeks after surgery. Subsequently, active range-of-motion (ROM) exercises for the ankle joint were initiated and gradually advanced to gentle passive ROM exercises, including inversion. Patients were encouraged to perform weight-bearing ambulation with an elastic ankle bandage as tolerated, and full weight-bearing gait was allowed 4 weeks after surgery. It was recommended that regular rehabilitation sessions supervised by a physiotherapist be conducted twice weekly for at least 8 weeks postoperatively. The rehabilitation regimen included peroneal muscle strengthening (eccentric–contraction eversion exercises), tandem stance, single-limb stance, single-limb stance with ball toss, wobble board exercise, quadrant hop drills, and treadmill running. Return to sport was made in accordance with the clinical guideline suggested by Song et al. [13] and was permitted no sooner than 8 weeks postoperatively. At each follow-up visit, proprioceptive-oriented exercises aimed at reducing postural sway and enhancing postural control ability were continuously encouraged. The full duration of proprioceptive-oriented rehabilitation was closely monitored using the modified Romberg test (single-leg stance test with eyes closed). All the patients were encouraged to improve their balance retention time, from when they placed their non-stance leg on the floor to when they had a feeling of being unable to maintain a one-leg standing position, and reported the best record at follow-up visit.

### 2.3. Assessment of Patient-Reported Clinical Outcomes

The changes of clinical outcomes before and after surgery were periodically evaluated using the Foot and Ankle Outcome Score (FAOS) [14] and the Foot and Ankle Ability Measure (FAAM) [15]. The reliability of these patient-reported outcome measures has been validated for patients with lateral ankle instability [14,15]. The FAOS questionnaire contains 5 distinct subscales evaluating pain (9 questions), other symptoms (7 questions), function in daily living (17 questions), function in sport and recreation (5 questions), and foot- and ankle-related quality of life (4 questions). The FAAM questionnaire consists of 2 subscales, evaluating activities of daily living (21 questions) and sports activities (8 questions). The total subscale scores were calculated and normalized to a 100-point scale. A score of 100 reflects optimal clinical status without symptoms, while a score of 0 signifies the poorest function and severe symptoms.

### 2.4. Assessment of Mechanical Ankle Stability

Mechanical ankle stability was periodically assessed before and after surgery using both physical examination and stress radiography. A senior surgeon consistently performed the manual varus and anterior drawer stress tests, comparing results to the contralateral (unaffected) ankle. Varus and anterior drawer stress radiographs of the ankle were obtained with a Telos device (Telos GmbH, Marburg, Germany), utilizing a standardized load of 150 Newton. Three independent researchers measured the talar tilt angle and the degree of anterior talar translation on a digital PACS imaging system. Each measurement task was carried out twice, and the average value was used for analysis.

### 2.5. Assessment of Peroneal Strength

Concentric and eccentric muscle strengths for eversion were periodically measured using the Biodex-II isokinetic dynamometer (Biodex Medical Systems, Shirley, NY, USA). Peak torque normalized to body weight, as well as total work were measured at an angular velocity of 60°/s. All assessments were administered by the same physical therapist following a standardized examination protocol. Each evaluation was conducted two times with a 5 min interval between tests, and the results were averaged. Peak torque represented the highest force generated at any point during five consecutive repetitions at 60°/s, serving as an indicator of muscle strength. Total work indicated the average amount of force produced during repetitive motions and represented muscle endurance.

### 2.6. Assessment of Static and Dynamic Postural Control Ability

**Figure 2:**
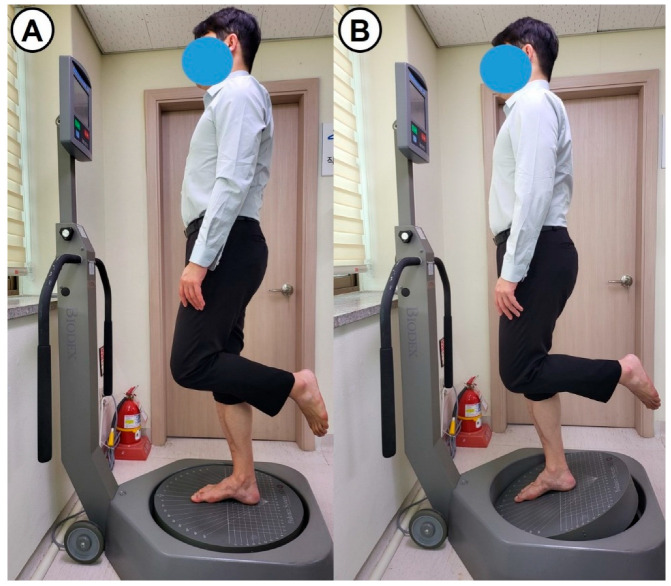
Photographs show the quantitative evaluation of (**A**) static and (**B**) dynamic postural control ability using Biodex posturography. Participants stand barefoot with one leg on the platform.

Static and dynamic postural control abilities were periodically evaluated using Biodex posturography (Biodex Medical Systems, Shirley, NY, USA), which is recognized for its reliability in balance assessment [16]. Patients received real-time visual biofeedback on a monitor displaying shifts in their center of gravity as they maintained a one-leg standing posture with eyes open. This task was performed during automated changes in platform tilt and rotation, progressing from level 8 (most stable) to level 1 (most unstable) over 20 s (Figure 2). The balance platform moved up to 20° of planar slope in a 360° range of motion. Under the supervision of the same researcher, both the static (on a fixed platform) and dynamic (on a mobile platform) stability tests were repeated two times with an interval of 3 min, and the measurements were averaged. The anterior–posterior stability index (APSI), medial–lateral (MLSI) stability index, and overall stability index (OSI) were recorded as quantitative measures related to postural stability. Each index reflected the horizontal deviation (fluctuation) from the center of pressure (COP), with higher index values indicating increased movement away from the individual’s center of gravity (poor balance).

### 2.7. Statistical Analysis

The statistical analysis was performed using SPSS 23.0 (SPSS Inc., IBM Company, Chicago, IL, USA), and a *p* value ≤ 0.05 with a confidence interval of 95% was set to the statistical level of significance. A normal distribution of all the collected data was confirmed with Shapiro–Wilks and Kolmogorov–Smirnov normality tests. The Mann–Whitney U test was used to compare the patient-reported clinical outcomes, mechanical ankle stability, peroneal strength, and static and dynamic postural control ability between the return group and non-return group. Categorical data, such as patient demographics and complication rates, were compared using the Fisher exact test. The changes between before and after surgery in the same individuals were compared using the Wilcoxon signed-rank test. Multivariate logistic regression analysis was performed to assess which variables are independent predictors of failure to return to sports activity. On power analysis to determine the appropriate sample size, we calculated that allocation of 11 patients into each group would provide 80% power to compare the validated functional outcome measure (FAOS). The 95% confidence interval (a type-I error rate of 0.05) was used to analyze whether the difference in sports activity subscale of the FAOS at 3 years postoperative was within a margin of noninferiority. The set margin of noninferiority was −10 points (a delta of 10 points in the FAOS), and the estimated dropout rate was 5%. The margin of noninferiority and the dropout rate were determined on the basis of the clinical data of previous studies. Eventually, the sample size required to make statistically significant results was 11~12 patients in each group.

## 3. Results

### 3.1. Comparison of Demographic and Clinical Characteristics

No statistically significant differences were observed in the demographic and clinical characteristics between the return and non-return groups (Table 1).

### 3.2. Comparison of Patient-Reported Clinical Outcomes

Both the return and non-return groups showed significant improvements in FAOS and FAAM scores compared to their preoperative values (Table 2). When examining each subscale, there were significant differences in FAOS pain (94.7 points in the return group vs. 85.1 points in the non-return group; *p* = 0.004) and FAOS sports (91.2 vs. 78.8 points; *p* < 0.001) subscales. In addition, the FAAM sports activity subscale showed a significant difference between the groups (90.5 vs. 77.4 points; *p* < 0.001).

### 3.3. Comparison of Postoperative Complications

With the exception of the recurrence rate of ankle instability, the postoperative complication rates did not differ significantly between the return and non-return groups (Table 3). The recurrence rate of mechanical ankle instability was significantly higher in the non-return group (36.4%) compared to the return group (3.9%) (*p* < 0.001).

### 3.4. Comparison of Mechanical Ankle Stability

Postoperative periodic physical examinations (stress test) revealed mechanical ankle instability as unstable as that prior to surgery in 2 patients in the return group and 4 patients in the non-return group. Periodic stress radiographic examinations demonstrated significant improvements in both talar tilt angle and anterior talar translation in both groups compared to preoperative values (Table 4). No significant differences were detected between the return and non-return groups in stress radiographic measurements.

### 3.5. Comparison of Isokinetic Peroneal Strength

Both the return and non-return groups showed significant improvements in concentric and eccentric muscle strength for eversion compared to prior to surgery (Table 5). There were no significant differences in concentric and eccentric peroneal strength between the return and non-return groups.

### 3.6. Comparison of Static and Dynamic Postural Control Ability

Static postural control abilities did not show significant improvement in either the return or non-return group when compared to preoperative assessments (Table 6). Additionally, no significant differences were identified in APSI, MLSI, and OSI between the two groups.

The return group demonstrated a significant improvement in dynamic postural control ability compared to prior to surgery, whereas the non-return group demonstrated no significant improvement (Table 7). An analysis of individual stability indices revealed significant differences in APSI (0.94 in the return group vs. 1.95 in the non-return group; *p* < 0.001), MLSI (0.73 vs. 1.58; *p* = 0.041), and OSI (1.41 vs. 2.33; *p* = 0.002).

### 3.7. Comparison Between Professional Athletes and Amateur (Recreational Level) Participants

All (11 patients) professional or junior-level athletes were able to return to their preinjury level of sports activity, whereas 11 (21.6%) out of a total of 51 patients with a recreational or amateur level of sports activity failed to return to preinjury level of sports activity (*p* < 0.001). Statistically significant differences were observed in FAOS sports (95.1 in the professional group vs. 84.6 points in the amateur group; *p* = 0.001) and FAAM sports subscales (94.2 vs. 83.8 points; *p* = 0.004). Recurrence of mechanical instability occurred in 1 patient (9.1%) in the professional group and in 5 patients (9.8%) in the amateur group, showing no significant difference. No significant differences were detected in measurements of stress radiography or peroneal strength. A posturography assessment revealed significant differences in static postural control ability (overall stability index: 0.92 in the professional group vs. 1.37 in the amateur group; *p* = 0.039) and dynamic postural control ability (1.13 vs. 1.98; *p* = 0.001).

### 3.8. The Risk Factors Associated with Failure to Return to the Preinjury Level of Sports Activity

Multivariate logistic regression analyses of variables were significantly different between the return and non-return groups and identified several risk factors associated with a failure to return to the preinjury level of sports activity. Patients with an FAOS pain score < 90 demonstrated a higher risk of failure to RTS compared to those with an FAOS pain ≥ 90 (odds ratio 8.66; *p* < 0.001) (Table 8). Patients with recurrence of mechanical instability were at a greater risk of failure to RTS than those without recurrent instability (odds ratio 13.99; *p* < 0.001). Additionally, patients with inadequate dynamic postural control ability > overall stability index 1.8 had a greater risk of failure to RTS than those with OSI ≤ 1.8 (odds ratio 4.21; *p* = 0.012).

## 4. Discussion

This comparative study reports specific factors that make the difference between patients with and without return to preinjury level of sports activity in 62 patients with a periodic follow-up of 3 years after anatomical ligament repair for CLAI. The most critical finding is that residual pain, recurrence of mechanical instability, and insufficient recovery of dynamic postural control ability are associated with the return to preinjury level of sports activity after the MBP. These findings provide valuable information to guide the functional rehabilitation process aimed at facilitating a return to sports activity following surgery.

Successful return to sports activity is a key factor for evaluating functional outcomes and influences patient satisfaction following operative treatment for CLAI. However, there is limited literature addressing the underlying reasons for delayed or unsuccessful recovery to the previous level of sports activity. The reduction in return to sports activity after operative treatment for CLAI is widely believed to result from multiple factors, many of which are challenging to measure objectively [11,12,17,18]. Sigonney et al. have suggested that increased patient perception and fear of re-injury is a critical cause of decreased RTS [19]. Substantial agreement exists that intra- or extraarticular lesions associated with acute lateral ligament injury or CLAI are among the leading causes [5,10,12,18]. In the present study, we excluded patients exhibiting concomitant advanced osteochondral lesions that required microfracture or osteochondral transplantation. Our operative experience indicates that such advanced osteochondral lesions are frequently associated with more persistent symptoms and diminished functional activity.

Postoperative recurrence of instability is recognized as an additional contributing factor. A long-term follow-up study after the MBP found that around 26% of patients had ceased active sports participation and 16% transitioned to sports activities at a lower level [7]. In that study, the most critical cause leading to failure to RTS was recurrence of mechanical and functional ankle instability. Similarly, Bridgman et al. documented that patients with recurrent ankle instability were more likely to discontinue sports participation [20]. Our results show a markedly lower rate of recurrent mechanical instability in the RTS group (3.9%) compared with the non-return group (36.4%), underscoring the impact of instability recurrence on RTS.

With respect to lateral ligament repair for acute grade-III injuries (characterized by complete rupture of the ATFL and the CFL with substantial instability), a meta-analysis by Kerkhoffs et al. found that surgical treatment was superior to conservative treatment in terms of return to preinjury level of activities, rate of recurrence, chronic pain, and subjective or functional instability [21]. Hong et al. reported that the median time to return to preinjury level of sports was 69 days in elite athletes [10]. White et al. reported that the median time for RTS was 11 weeks in professional athletes [8]. Their study identified that the major contributors to delayed return to training and competition were concomitant injuries, such as osteochondral lesions or deltoid ligament involvement. Goru et al. further indicated that associated intra-articular or extra-articular lesions were a primary reason for delayed recovery after surgical management [18]. Patients with CLAI are also predisposed to a variety of intra-articular pathologies secondary to repeated sprain episodes [1,3,22]. Even when appropriate interventions for these concurrent pathologies are implemented, such patients may still experience ongoing symptoms and obstacles in resuming sports participation. Raja et al. reported that postoperative residual pain did not account for diminished RTS rates [23]. Conversely, the present study identified residual pain as one of the determinants affecting RTS after anatomical ligament repair for CLAI. Patients who returned to preinjury level of sports activity (return group) exhibited significantly superior pain scores compared to the non-return group. Therefore, more comprehensive diagnostic approaches and tailored treatments are recommended for individuals unable to RTS due to persistent, long-term postoperative pain.

Postural control ability is understood to depend on visual, vestibular, and proprioceptive inputs [24]. Multiple individual factors, such as proprioception, nerve conduction velocity, postural reflex, joint range of motion, and muscle strength, contribute to postural control ability of the ankle joint [25]. The mechanoreceptors in the ligaments and capsule of the ankle joint serve as crucial sources of afferent proprioceptive information. Patients with CLAI frequently report deficient postural control attributable to damage to proprioceptors resulting from repeated sprains [17,25]. A systematic review reported that a deficit of postural control ability was associated with increased risk of ankle sprain [26]. In this study, inadequate recovery of dynamic postural control ability was among the factors influencing RTS following the MBP for CLAI. Those achieving a return to their preinjury sports participation demonstrated notably higher stability index scores compared to those who did not return, whereas there was no significant difference in the assessment of static postural control ability between the two groups. Static postural stability measured by posturography may lack sensitivity in distinguishing between CLAI patients who did and did not achieve return to preinjury level of sports participation [9,26].

Regarding the impact of demographic factors on RTS, Lee et al. found no significant differences in age, sex, body mass index (BMI), grade of instability, presence of subfibular ossicle, and preoperative functional evaluation score (American Orthopedic Foot and Ankle Society score) between the early RTP and late RTP groups [11]. In contrast, Li et al. reported that each decade of age increased the risk of RTS failure by 6%, and a 5 kg/m^2^ increase in BMI raised the risk of RTS failure by 4% [12]. They also indicated that no significant difference existed in RTS rates when comparing arthroscopy to open surgery, repair versus reconstruction, and early versus late weight-bearing in the rehabilitation protocol [12]. Additionally, Bouveau et al. reported a significant association between lower BMI and achieving preinjury level of RTS [27]. In the present study, no significant differences were observed in the demographic and clinical characteristics between the return and non-return groups.

A recent systematic review focusing on studies of ankle lateral ligament reconstruction in professional athletes concluded that approximately 89% of the patients returned to their preinjury level of sports activity, 2% returned at a lower level, and the remaining 9% did not return to their preinjury level of sports [18]. Moreover, the mean time to resume physical training and sports was found to be around 16 weeks postoperatively. Cho et al. reported the average period to return to exercise following the ligament reattachment procedure using the suture bridge technique in athletes: approximately 8.4 weeks for jogging, 10.5 weeks for jumping, and 12.5 weeks for spurt running [28]. According to Lee et al., the mean length of time to RTS after the MBP in elite athletes was 1.9 months for return to personal training, 2.9 months for return to team training, and 3.9 months for return to competitive play (official game participation) [11]. Their findings show that 83.3% of athletes resumed play at 4 months, with a complete 100% return at 8 months postoperatively. With long-term follow-up (a mean of 10.6 years) after MBP, Lee et al. reported that 28 of 30 patients could participate in their preinjury level of activities [29]. In the case of arthroscopic surgery for CLAI, Bouveau et al. reported that the patient’s preoperative motivation to RTS was significantly correlated with both the rate of and the time to RTS [27].

As one of the possible factors affecting RTS after operative treatment for CLAI, the substantial variation in both surgical techniques and rehabilitation protocols should be acknowledged. With a concern for early elongation of the repaired ligaments, various modifications in operative procedure and rehabilitation strategy have been ongoing [30,31,32,33,34]. A current concepts review by Lan et al. indicated that MBP with suture-tape augmentation may allow a faster and more intensive rehabilitation protocol without compromising the restored stability [31]. Coetzee et al. reported that MBP augmented with suture tape led to low rates of recurrent instability and rapid RTS (average 84 days after surgery) [32]. Kulwin et al. reported that MBP with suture-tape augmentation enabled a successful accelerated rehabilitation process compared to MBP alone and resulted in an earlier return to the preinjury level of activity (average 13.3 vs. 17.5 weeks) [30]. In their study, 12.5% of patients treated with MBP and 3.5% of those with MBP augmented with suture tape failed to return to preinjury activity level by 26 weeks postoperatively. Regardless of the surgical techniques for CLAI, clinicians should consider the patient-specific best timing for RTS after surgery. Lee et al. reported that dynamic postural stability and neuromuscular control were still significantly reduced at 12 weeks (a commonly recommended time for RTS after MBP) when compared to the normal control group [9].

This study has several limitations. The first point is the recruitment of various participants with heterogenous neuromuscular functionality for sports activity. This study eventually included a small proportion of professional or junior-level athletes. Prior to operative treatment, most patients participated in recreational or amateur-level sports activity. Since RTS is a gradual process following surgery and the expected performance differs across individuals, the compliance and willingness (motivation) to return during the rehabilitation period may vary substantially among the patients [12]. Consequently, data from the current study may only be applicable to the general population rather than high-demand athletes. Second, there was heterogeneity in the details of the MBP technique (open or arthroscopic approach, types and numbers of suture anchors, insertion point of suture anchors, the use of the CF ligament or inferior extensor retinaculum, et al.) across surgeons, which could introduce bias and affect the generalizability of the results. Third, this study included an equivalent rehabilitation training supervised by physiotherapist two times per week up to at least 8 weeks after surgery. Although continued peroneal strengthening and proprioceptive-oriented exercises were encouraged at every follow-up visit, substantial variation in patient compliance was likely after the initial 8-week period. Fourth, classification of RTS was made based on patient self-report instead of standardized tools or objective performance metrics. Standardized and validated tools [19,35,36] related to RTS have been reported to be useful to evaluate various factors including the level of sport activity and psychological readiness before returning to sport. Fifth, this study was performed with a relatively small sample size, including the non-return group comprising only 11 patients. The small sample size may have an impact on both the statistical power and the potential for false-negative findings. In addition, statistical analysis was performed without any correction for multiple testing. The lack of adjustment for multiple comparisons is a statistical limitation in this study. Finally, the lack of evaluation for psychological factors is another limitation of this study. Fear of re-injury, psychological withdrawal, and psychological impatience for a quick recovery can significantly affect RTS. Consequently, the influence of these psychological variables should be systematically assessed in future studies with higher levels of evidence.

## 5. Conclusions

This prospective series of patients who underwent the MBP for CLAI has demonstrated that residual pain, recurrence of mechanical instability, and insufficient recovery of dynamic postural control ability are associated with the return to preinjury level of sports activity. It is essential to develop effective treatment strategies that address these factors during the rehabilitation process to facilitate successful return to sports activity after surgery.

## Figures and Tables

**Table 1:** Comparison of demographic and clinical characteristics (Mann–Whitney U test and Fisher exact test).

Demographic Information *	Return Group (n = 51)	Non-Return Group (n = 11)	*p*-Value
Sex, n (%)			
Male	31 (60.8%)	7 (63.6%)	0.855
Female	20 (39.2%)	4 (36.4%)	0.834
Age at surgery (years)	26.8 ± 8.7	27.4 ± 9.1	0.901
Duration of instability (months)	35.1 ± 18.3	32.5 ± 16.8	0.726
Body mass index (kg/m^2^)	24.2 ± 8.3	24.4 ± 8.5	0.928
Combined intra-articular lesion, n (%)			
Osteochondral lesion	9 (17.6%)	2 (18.2%)	0.952
Synovitis	29 (56.9%)	7 (63.6%)	0.561
Loose body	4 (7.8%)	1 (9.1%)	0.784
Impinging bony spur	2 (3.9%)	0 (0%)	0.635
Subfibular ossicle, n (%)	11 (21.6%)	2 (18.2%)	0.691
Sports activity level, n (%)			
Professional (competition)	9 (17.6%)	2 (18.2%)	0.867
Recreational (regular)	28 (54.9%)	7 (63.6%)	0.215
Recreational (occasional)	14 (27.5%)	2 (18.2%)	0.124
High-demand laborer, n (%)	15 (29.4%)	4 (36.4%)	0.371

* Data are represented as mean ± standard deviation.

**Table 2:** Comparison of the patient-reported clinical outcomes (Mann–Whitney U test and Wilcoxon signed-rank test).

Subscales		Preoperative	PO 3 Years	*p*-Value ^†^
FAOS *				
Pain	Return group	78.2 ± 18.5	**94.7 ± 5.3**	**<0.001**
	Non-return group	77.5 ± 17.3	**85.1 ± 10.7**	0.115
Other symptoms	Return group	73.4 ± 16.9	92.8 ± 7.1	**<0.001**
	Non-return group	75.1 ± 16.8	90.9 ± 8.9	**<0.001**
ADL	Return group	64.1 ± 19.1	92.6 ± 7.3	**<0.001**
	Non-return group	63.5 ± 18.6	91.3 ± 8.6	**<0.001**
Sports activity	Return group	38.2 ± 17.1	**91.2 ± 8.4**	**<0.001**
	Non-return group	39.4 ± 16.9	**78.8 ± 15.9**	**<0.001**
Quality of life	Return group	61.5 ± 19.7	95.8 ± 4.1	**<0.001**
	Non-return group	60.9 ± 18.6	92.5 ± 7.4	**<0.001**
Total scores	Return group	63.1 ± 17.7	93.4 ± 6.4	**<0.001**
	Non-return group	63.3 ± 17.9	87.7 ± 10.8	**<0.001**
	***p*-value ^‡^**	0.981	0.204	
FAAM *				
Daily activity	Return group	74.5 ± 17.2	95.1 ± 4.8	**<0.001**
	Non-return group	73.2 ± 18.1	92.9 ± 6.9	**<0.001**
Sports activity	Return group	44.6 ± 19.4	**90.5 ± 8.9**	**<0.001**
	Non-return group	43.4 ± 20.1	**77.4 ± 16.8**	**<0.001**
Total scores	Return group	59.6 ± 18.3	92.8 ± 6.7	**<0.001**
	Non-return group	58.3 ± 18.7	85.2 ± 11.5	**<0.001**
	***p*-value ^‡^**	0.835	0.116	

Abbreviation: FAOS, Foot and Ankle Outcome Score; FAAM, Foot and Ankle Ability Measure; PO, postoperative; ADL, activity of daily living. Statistically significant values are indicated in bold. * Data are represented as scores (mean ± standard deviation) changed on the basis of 100 points. ^†^ Comparison between preoperative and 3 years postoperatively (Wilcoxon signed-rank test). ^‡^ Comparison of total scores between return and non-return groups (Mann–Whitney U test).

**Table 3:** Comparison of postoperative complications (Fisher exact tests).

Complication, n (%)	Return Group (n = 51)	Non-Return Group (n = 11)	*p*-Value
Superficial wound infection	1 (1.9%)	0 (0%)	0.924
Delayed wound healing	3 (5.9%)	1 (9.1%)	0.547
Superficial peroneal nerve injury	2 (3.9%)	1 (9.1%)	0.351
Skin irritation by suture materials	1 (1.9%)	0 (0%)	0.924
Stiffness (ROM limitation > 10°)	1 (1.9%)	0 (0%)	0.924
Recurrence of ankle instability	2 (3.9%)	4 (36.4%)	**<0.001**

Abbreviation: ROM, range of motion. Statistically significant values are indicated in bold.

**Table 4:** Comparison of mechanical ankle stability evaluated with stress radiographs (Mann–Whitney U test and Wilcoxon signed-rank test).

Stress Radiographs	Preoperative	PO 3 Years	*p*-Value ^†^
Talar tilt angle (°) *			
Return group	15.5 ± 7.9	2.9 ± 1.5	**<0.001**
Non-return group	14.9 ± 7.5	3.8 ± 1.7	**<0.001**
***p*-value ^‡^**	0.809	0.216	
Anterior talar translation (mm) *			
Return group	14.8 ± 7.1	4.5 ± 2.4	**<0.001**
Non-return group	14.1 ± 6.8	5.1 ± 2.8	**0.001**
***p*-value ^‡^**	0.745	0.796	

Abbreviation: PO, postoperative. * Data are represented as mean ± standard deviation. ^†^ Comparison between preoperative and 3 years postoperatively (Wilcoxon signed-rank test). ^‡^ Comparison between return and non-return groups (Mann–Whitney U test). Statistically significant values are indicated in bold.

**Table 5:** Comparison of peroneal strength evaluated with isokinetic dynamometer (Mann–Whitney U test and Wilcoxon signed-rank test).

Strength for Eversion *	Preoperative	PO 3 Years	*p*-Value ^†^
Concentric peak torque (Nm)			
Return group	10.2 ± 3.8	13.6 ± 4.9	**<0.001**
Non-return group	10.4 ± 3.9	13.1 ± 4.6	**<0.001**
***p*-value ^‡^**	0.915	0.574	
Eccentric peak torque (Nm)			
Return group	16.7 ± 6.5	22.2 ± 7.3	**<0.001**
Non-return group	17.1 ± 6.8	20.8 ± 6.9	**<0.001**
***p*-value ^‡^**	0.729	0.094	
Concentric total work (Nm)			
Return group	6.1 ± 2.6	7.8 ± 2.9	**0.021**
Non-return group	6.2 ± 2.5	7.3 ± 3.1	0.136
***p*-value ^‡^**	0.972	0.476	
Eccentric total work (Nm)			
Return group	10.3 ± 4.2	12.4 ± 4.7	**<0.001**
Non-return group	10.5 ± 4.4	12.1 ± 4.8	**0.005**
***p*-value ^‡^**	0.894	0.865	

Abbreviation: PO, postoperative; Nm, Newton-meter. Statistically significant values are indicated in bold. * Data are represented as mean ± standard deviation. ^†^ Comparison between preoperative and 3 years postoperatively (Wilcoxon signed-rank test). ^‡^ Comparison between return and non-return groups (Mann–Whitney U test).

**Table 6:** Comparison of static postural control ability (Mann–Whitney U test and Wilcoxon signed-rank test).

Biodex Posturography	Preoperative	PO 3 Years	*p*-Value ^†^
A–P stability index *			
Return group	1.37 ± 0.66	0.97 ± 0.48	0.489
Non-return group	1.42 ± 0.71	1.12 ± 0.55	0.665
***p*-value ^‡^**	0.933	0.842	
M–L stability index *			
Return group	0.96 ± 0.53	0.71 ± 0.34	0.585
Non-return group	1.04 ± 0.59	0.78 ± 0.41	0.521
***p*-value ^‡^**	0.882	0.918	
Overall stability index *			
Return group	1.75 ± 0.81	1.22 ± 0.54	0.437
Non-return group	1.81 ± 0.83	1.43 ± 0.65	0.549
***p*-value ^‡^**	0.941	0.795	

Abbreviation: PO, postoperative; A–P, anterior–posterior; M–L, medial–lateral. * Data are represented as mean ± standard deviation. ^†^ Comparison between preoperative and 3 years postoperatively (Wilcoxon signed-rank test). ^‡^ Comparison between return and non-return groups (Mann–Whitney U test).

**Table 7:** Comparison of dynamic postural control ability (Mann–Whitney U test and Wilcoxon signed rank test).

Biodex Posturography	Preoperative	PO 3 Years	*p*-Value ^†^
A–P stability index *			
Return group	1.98 ± 0.81	0.94 ± 0.41	**<0.001**
Non-return group	1.93 ± 0.79	1.95 ± 0.77	0.965
***p*-value ^‡^**	0.785	**<0.001**	
M–L stability index *			
Return group	1.66 ± 0.72	0.73 ± 0.34	**0.005**
Non-return group	1.63 ± 0.71	1.58 ± 0.69	0.905
***p*-value ^‡^**	0.971	**0.041**	
Overall stability index *			
Return group	2.43 ± 0.88	1.41 ± 0.62	**<0.001**
Non-return group	2.39 ± 0.86	2.33 ± 0.85	0.893
***p*-value ^‡^**	0.965	**0.002**	

Abbreviation: PO, postoperative; A–P, anterior–posterior; M–L, medial–lateral. Statistically significant values are indicated in bold. * Data are represented as mean ± standard deviation. ^†^ Comparison between preoperative and 3 years postoperatively (Wilcoxon signed-rank test). ^‡^ Comparison between return and non-return groups (Mann–Whitney U test).

**Table 8:** The risk factors associated with the failure to the return to preinjury level of sports activity (multivariate logistic regression analysis).

Potential Risk Factors	Return Group (n = 51)	Non-Return Group (n = 11)	Odds Ratio (95% CI)	*p*-Value
Residual pain *				
FAOS pain < 90	12 (23.5%)	8 (72.7%)	8.66 (1.98–18.4)	**<0.001**
FAOS pain ≥ 90	39 (76.5%)	3 (27.3%)		
Recurrence of instability *				
Yes	2 (3.9%)	4 (36.4%)	13.99 (3.84–27.1)	**<0.001**
No	49 (96.1%)	7 (63.6%)		
Dynamic postural control ability *				
OSI > 1.8	15 (29.4%)	7 (63.6%)	4.21 (1.06–11.8)	**0.012**
OSI ≤ 1.8	36 (70.6%)	4 (36.4%)		

Abbreviation: CI, confidence interval; FAOS, Foot and Ankle Outcome Score; OSI, overall stability index. Statistically significant values are indicated in bold. * Data at 3 years postoperatively.

## Data Availability

The data presented in this study are available in the article.

## Abbreviations

The following abbreviations are used in this manuscript:

MBP	Modified Broström procedure
FAOS	Foot and Ankle Outcome Score
FAAM	Foot and Ankle Ability Measure
CLAI	Chronic lateral ankle instability
RTS	Return to sports
